# Supplementary figures and images for: CD39 expression by regulatory T cells participates in CD8+ T cell suppression during experimental Trypanosoma cruzi infection
Source: PLoS Pathog. 2024 Apr 29;20(4):e1012191. doi: 10.1371/journal.ppat.1012191 (PMC11081507; doi:10.1371/journal.ppat.1012191)

**A**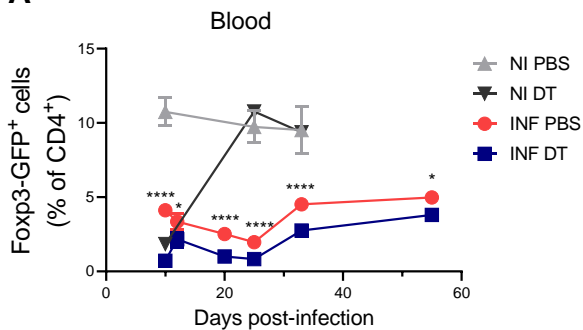**B**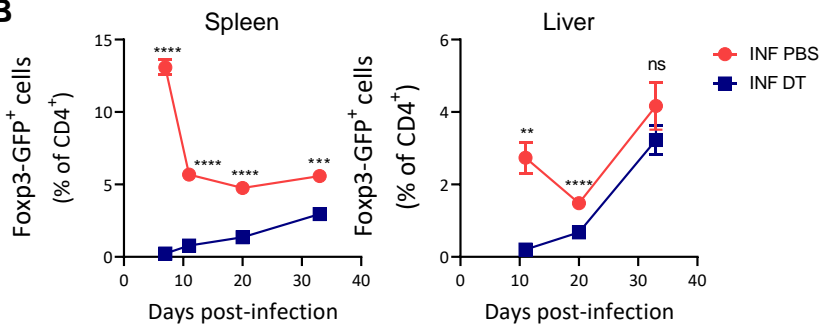**C**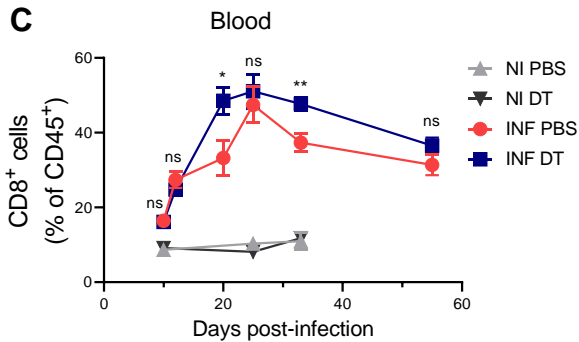**D**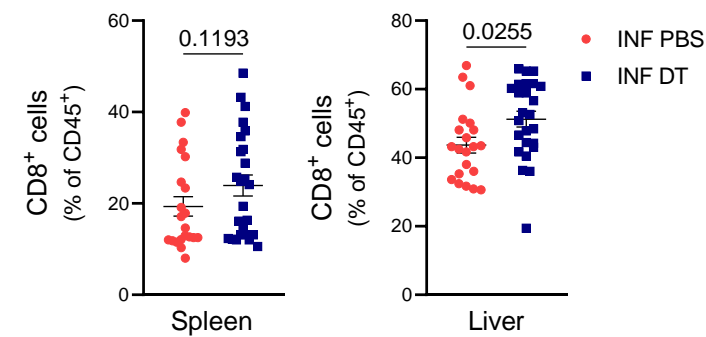**E**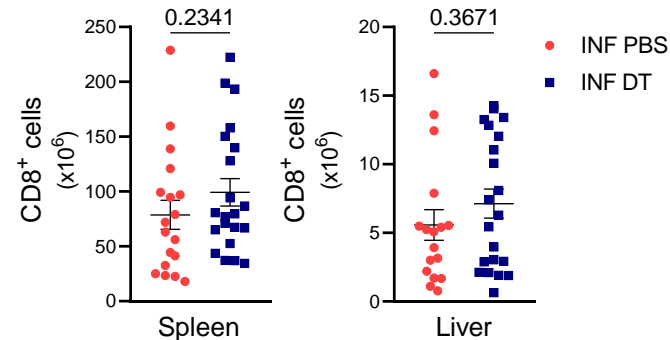

Supplement: S1 Fig — A-B) Kinetics analysis of Treg cells frequencies within gated CD4+ cells determined in blood (A), spleen and liver (B) from PBS or DT-treated, NI or T. cruzi INF DEREG mice. C-E) Frequency (C, D) and absolute numbers (E) of total CD8+ T cells determined at different time points in blood (C) and at 20 dpi in spleen and liver (D, E) of PBS or DT-treated, NI and INF DEREG mice. Data are presented as mean ± SEM. Data were collected from 1–7 independent experiments according to the tissue and dpi in (A-C) and from 4–5 independent experiments in (D-E). In (D-E) each symbol represents one individual mouse. A total of 2–36 mice per group were included. In (A) and (C) n = 2–3 for NI groups, n = 12–27 at 10 dpi, n = 7–14 at 12 dpi, n = 16–36 at 20 dpi, n = 6–10 at 25 dpi, n = 12–17 at 33 dpi, n = 5–7 at 55 dpi. In (B) n = 10–12 at 7 dpi, n = 3–4 at 11 dpi, n = 12–14 at 20 dpi, n = 4–5 at 33 dpi. Statistical significance was determined by Unpaired t test or Mann Whitney test, according to data distribution. Statistical analysis in A-C represents pairwise comparisons between INF PBS and INF DT groups. * P ≤ 0.05, ** P ≤ 0.01, *** P ≤ 0.001, **** P ≤ 0.0001 and ns = not significant. P values for pairwise comparisons at day 20 pi are indicated in the graphs. (PDF) [file ppat.1012191.s001.pdf]

**A**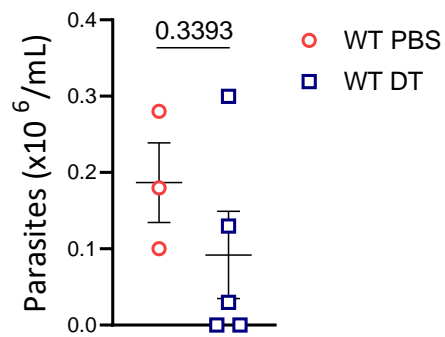**B**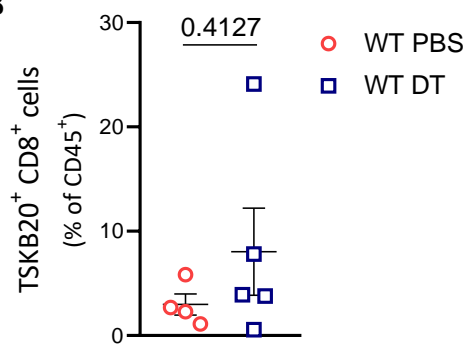**C**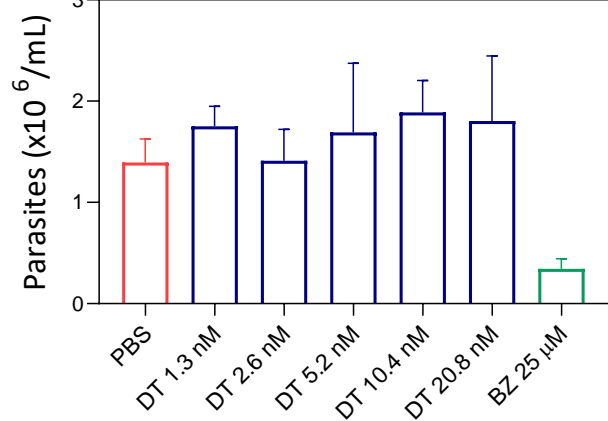**D**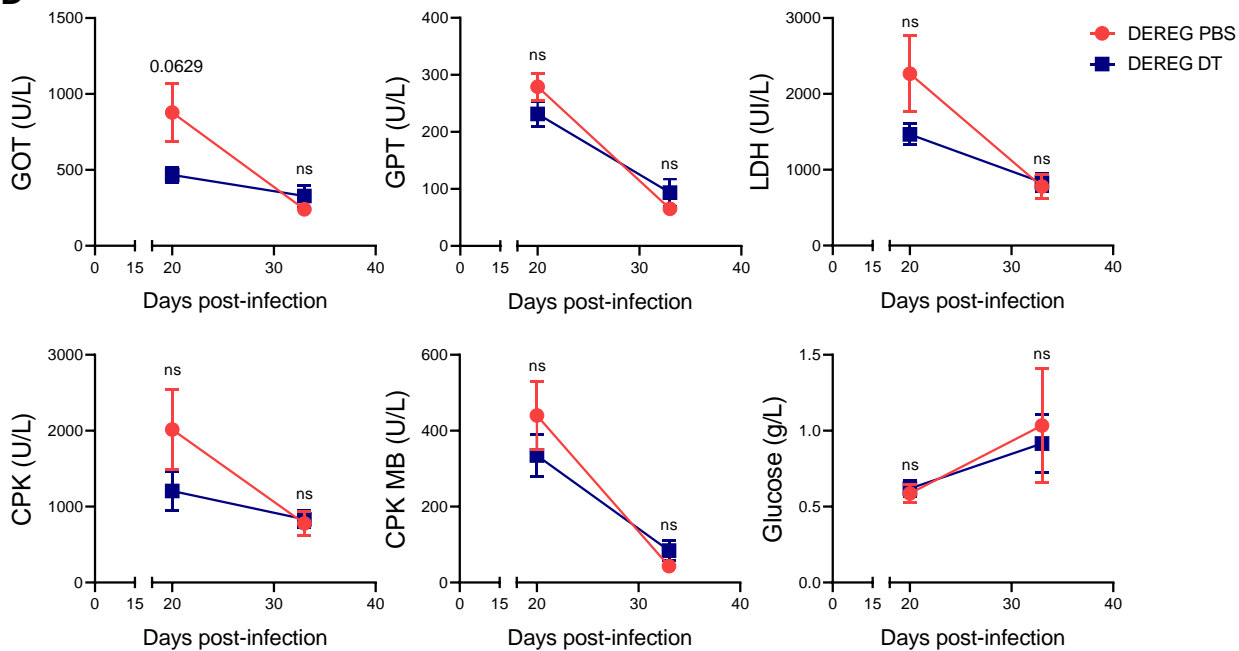

Supplement: S2 Fig — A-B) Parasite counts (A) and TSKB20-specific CD8+ T cell frequencies (B) in blood from PBS or DT-treated T. cruzi infected WT littermate mice at 20 dpi. Data were collected from 3 independent experiments and are presented as mean ± SEM. Statistical significance was determined by Mann Whitney test. C) Parasites numbers counted after 24 h of culture with PBS, increased doses of DT or benznidazole (BZ). Data are presented as mean ± SD of technical triplicates from 1 experiment. D) Treg cell depletion effect on tissue damage markers: activities of glutamate-oxalacetic transaminase (GOT), glutamate-pyruvate transaminase (GPT), lactate dehydrogenase (LDH), creatine phosphokinase (CPK), and creatine phosphokinase of muscle and brain (CPK MB), as well as Glucose concentration in plasma of PBS or DT-treated DEREG mice at days 20 and 33 pi. Data were collected from 6 independent experiments at 20 dpi (n = 20–26) and from 1 experiment at 33 dpi (n = 4–5). Data are presented as mean ± SEM. Statistical significance was determined by Unpaired t test for GOT, LDH, CPK, and CPK MB activities and Glucose concentration, and by Mann Whitney test for GPT activity, according to data distribution. P values for pairwise comparisons at day 20 pi are indicated in the graphs. ns = not significant. (PDF) [file ppat.1012191.s002.pdf]

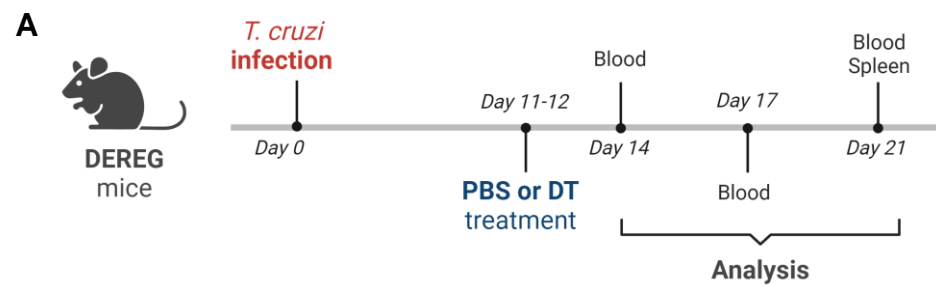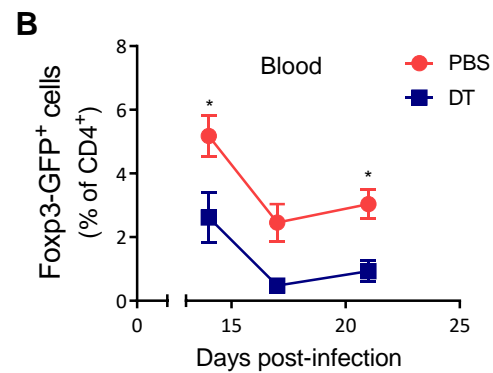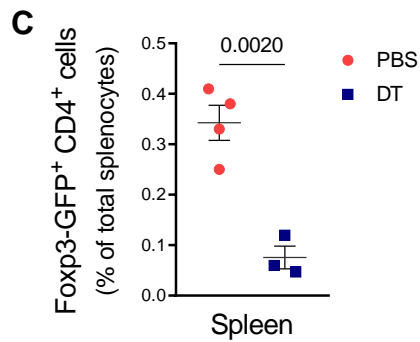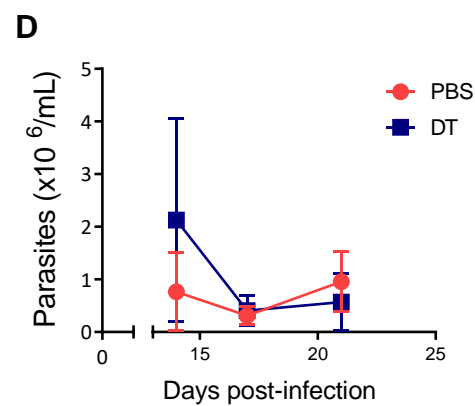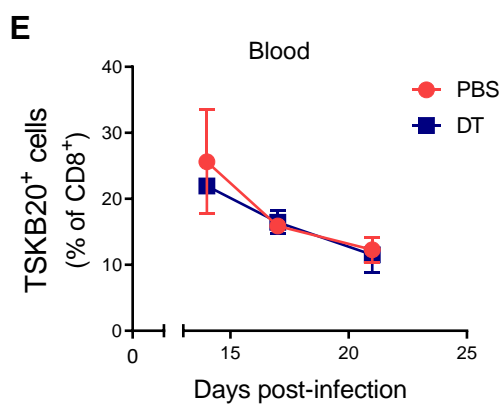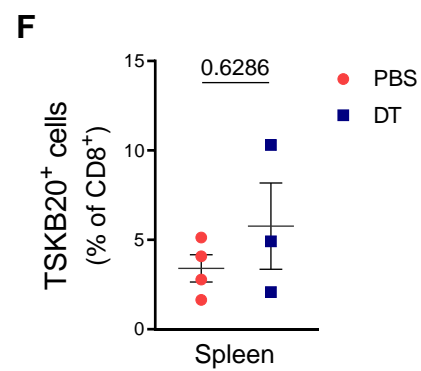

Supplement: S3 Fig — A) Experimental scheme for DT treatment (created with BioRender.com). B-C) Treg cell frequencies in blood at different dpi (B) and in spleen at day 21 pi (C) from T. cruzi-infected DEREG mice treated with PBS or DT on days 11 and 12 pi. D) Parasitemia levels from mice in (A). E-F) TSKB20-specific CD8+ T cell frequencies in blood at different dpi (E) and in spleen at day 21 pi (F) of mice in (A). All data are presented as mean ± SEM. Data were collected from 1–2 independent experiments. A total of 2–6 mice per group were included. Statistical significance was determined by Unpaired t test or Mann Whitney test, according to data distribution. P values for pairwise comparisons are indicated in the graphs. * P ≤ 0.05. (PDF) [file ppat.1012191.s003.pdf]

**A**

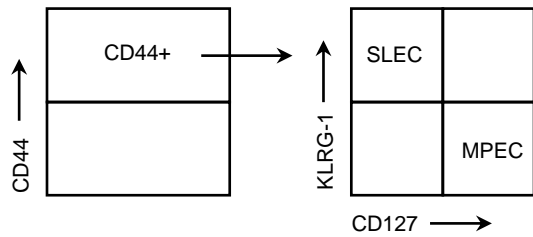

**B**

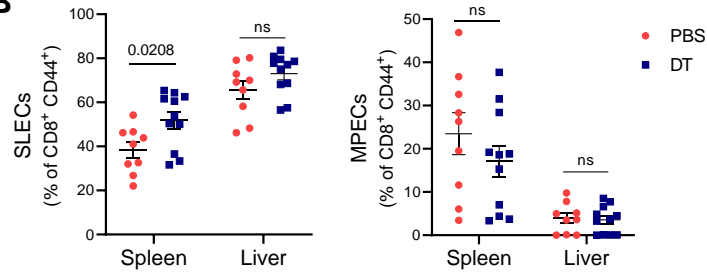

**C**

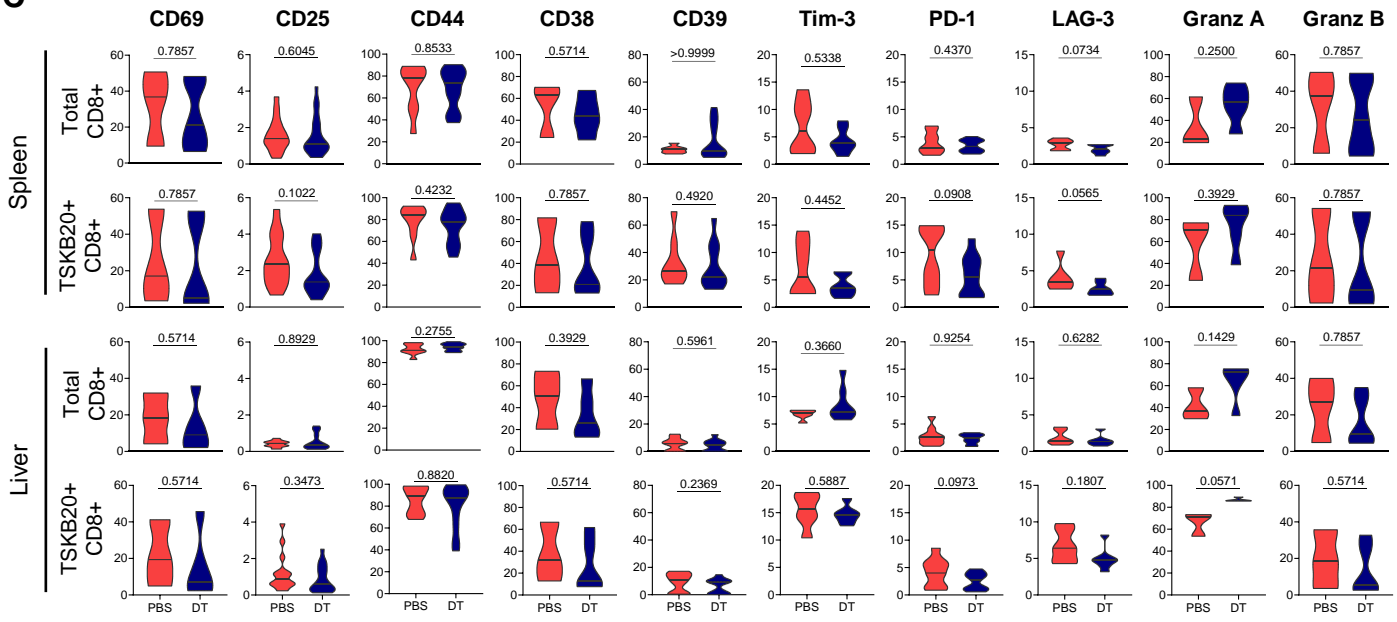

Supplement: S4 Fig — A) Gating strategy for evaluation of SLEC and MPEC subsets. B) Frequencies of SLEC (left) and MPEC (right) subsets within CD44+ gated CD8+ T cells from PBS or DT-treated DEREG mice at day 20 pi. Data were collected from 2 independent experiments and are presented as mean ± SEM. C) Comparison of the frequencies of cells expressing the indicated activation, exhaustion and functional markers in total and TSKB20-specific CD8+ T cells in the spleen and liver of PBS or DT-treated DEREG mice at day 20 pi. Data were collected from 1–4 independent experiments. Violin plots depict the distribution of frequency of 3–18 mice per group. Statistical significance was determined by Unpaired t test or Mann Whitney test, according to data distribution. P values for pairwise comparisons are indicated in the graphs. (PDF) [file ppat.1012191.s004.pdf]

**A**

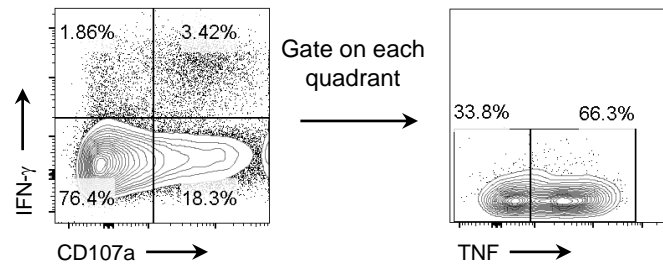

**B**

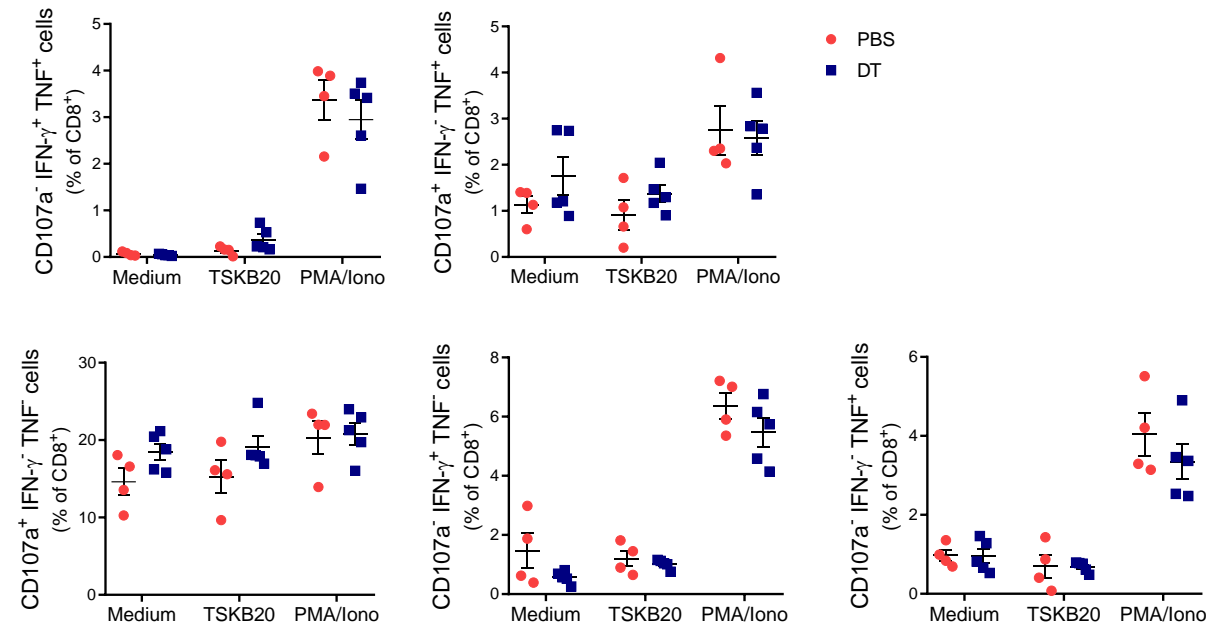

Supplement: S5 Fig — A) Gating strategy for assessing effector cytokine production and CD107a surface mobilization in gated CD8+ cells. B) Percentage of CD8+ T cells from the spleen of PBS or DT-treated DEREG mice at day 21 pi that exhibit different combinations of effector functions, including CD107a mobilization and/or IFN-γ and/or TNF production upon 5h of the indicated stimulation. Medium condition was used as a negative control, while PMA/Ionomycin (PMA/Iono) was used as a positive control for polyclonal CD8+ T cell stimulation. Similar results were obtained in 2 independent experiments. All data are presented as mean ± SEM. Each symbol represents one individual mouse. Statistical significance was determined by Mann Whitney test. (PDF) [file ppat.1012191.s005.pdf]

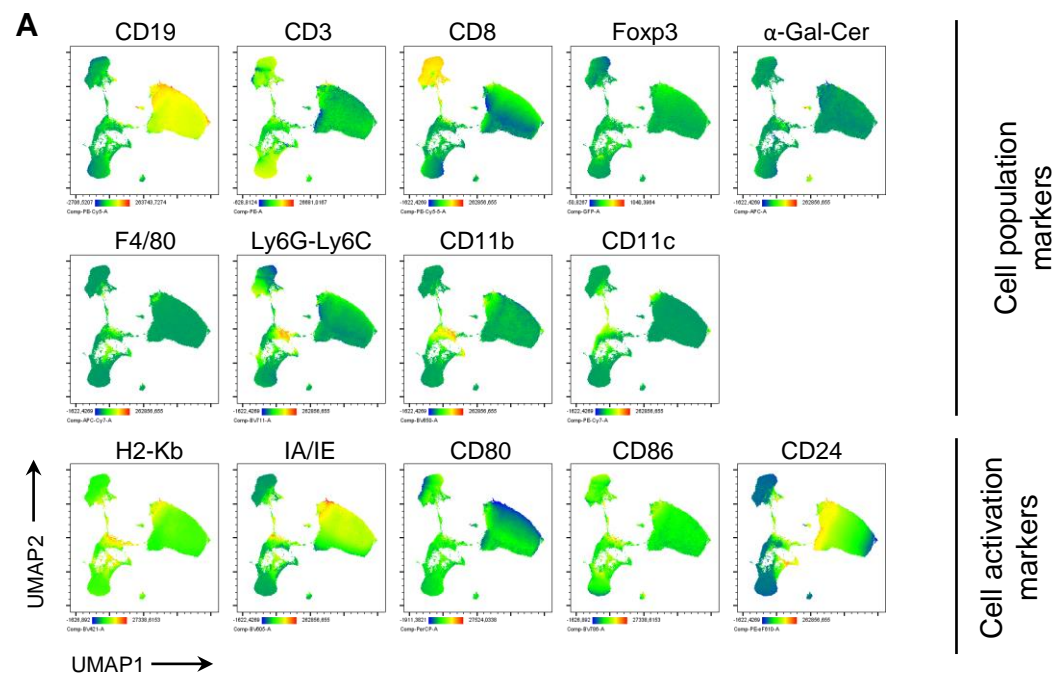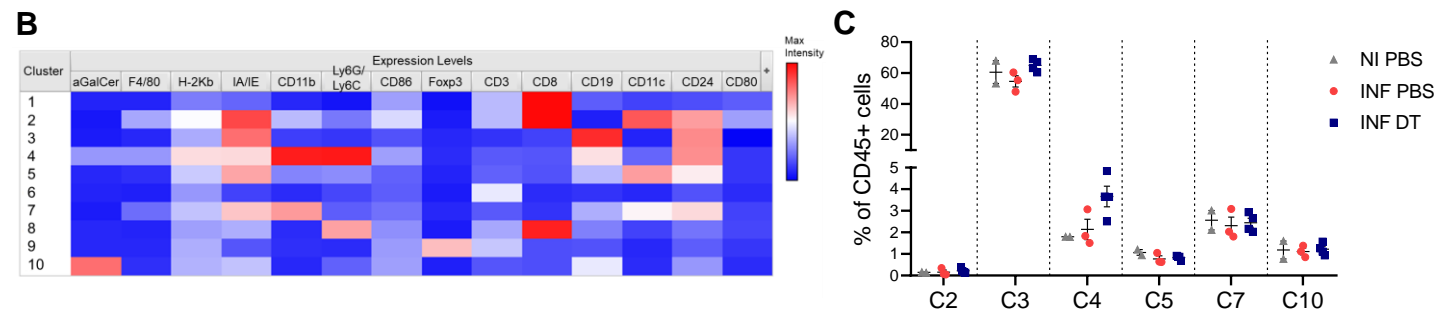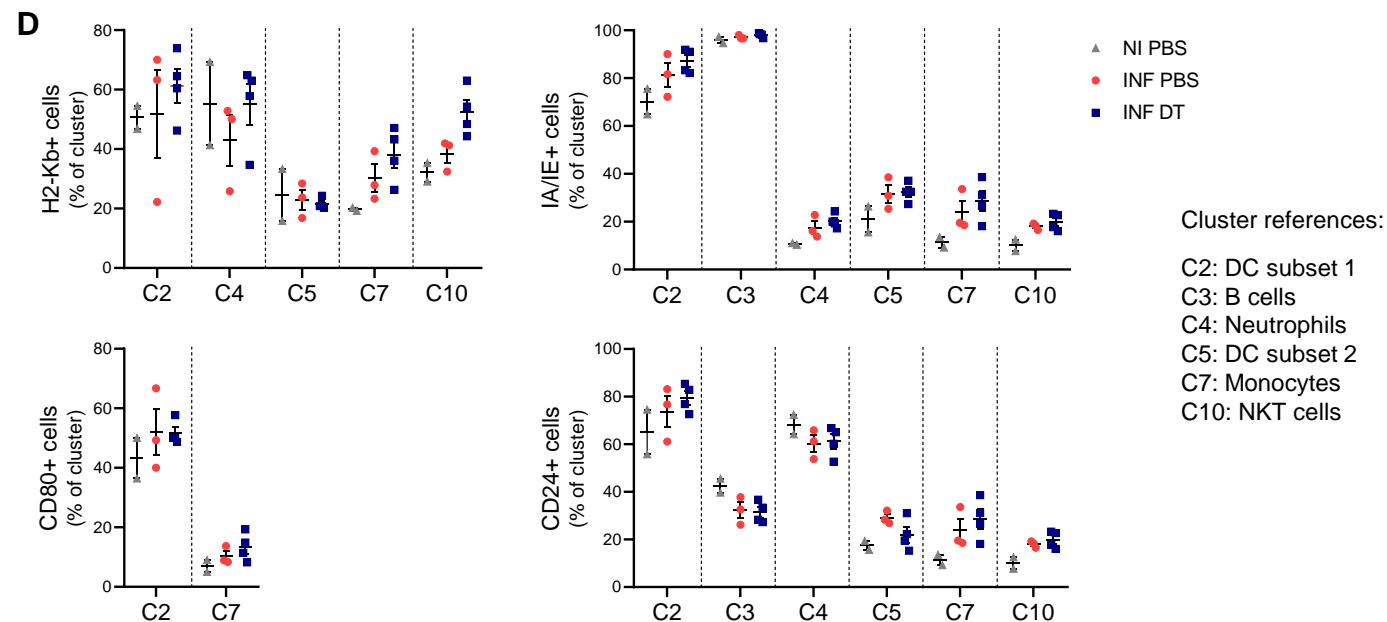

Supplement: S6 Fig — A) UMAP visualization for splenocytes expression of the different population and activation markers used in the flow cytometry panel for APC and innate cells characterization. Samples from the three experimental groups (NI PBS, INF PBS and INF DT) are shown together. B) Heat map showing the expression level of each marker in the different clusters. C) Frequencies of selected clusters in total leukocytes (CD45+ cells) from the spleen of PBS or DT-treated DEREG mice at day 7 pi and non-infected controls. D) Frequency of cells expressing the indicated markers in the different clusters defined in Fig 4A. Clusters without positive cells for the corresponding marker were excluded from the analysis. All data are presented as mean ± SEM. Each symbol represents one individual mouse. Similar results were obtained in 3 independent experiments. (PDF) [file ppat.1012191.s006.pdf]

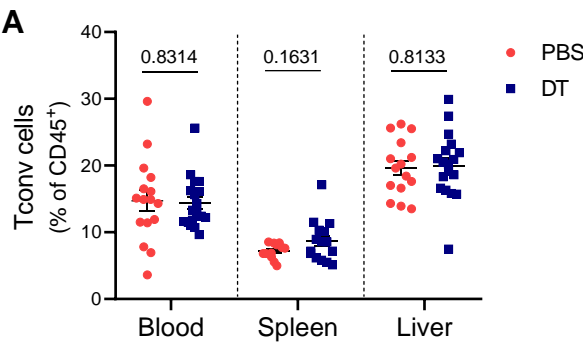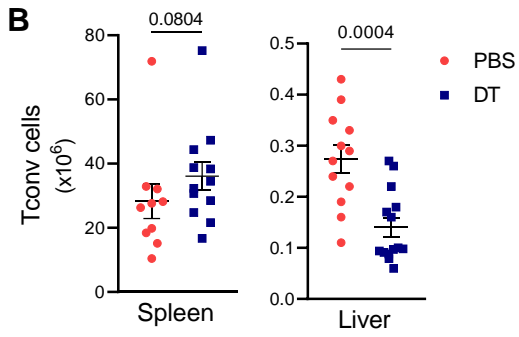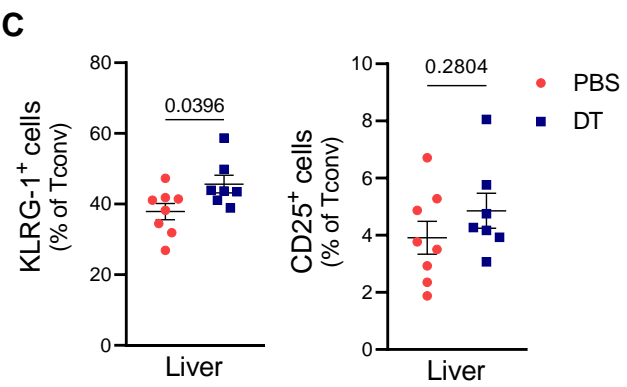

Supplement: S7 Fig — A-B) Frequencies (A) and absolute numbers (B) of Tconv cells in blood, spleen and liver from PBS or DT-treated DEREG mice at day 20 pi. C) Frequency of KLRG-1+ and CD25+ Tconv cells in the liver of PBS or DT-treated DEREG mice at day 11 pi. All data are presented as mean ± SEM. Each symbol represents one individual mouse. Data were pooled from 2–4 independent experiments. Statistical significance was determined by Unpaired t test or Mann Whitney test, according to data distribution. P values for pairwise comparisons are indicated in the graphs. (PDF) [file ppat.1012191.s007.pdf]

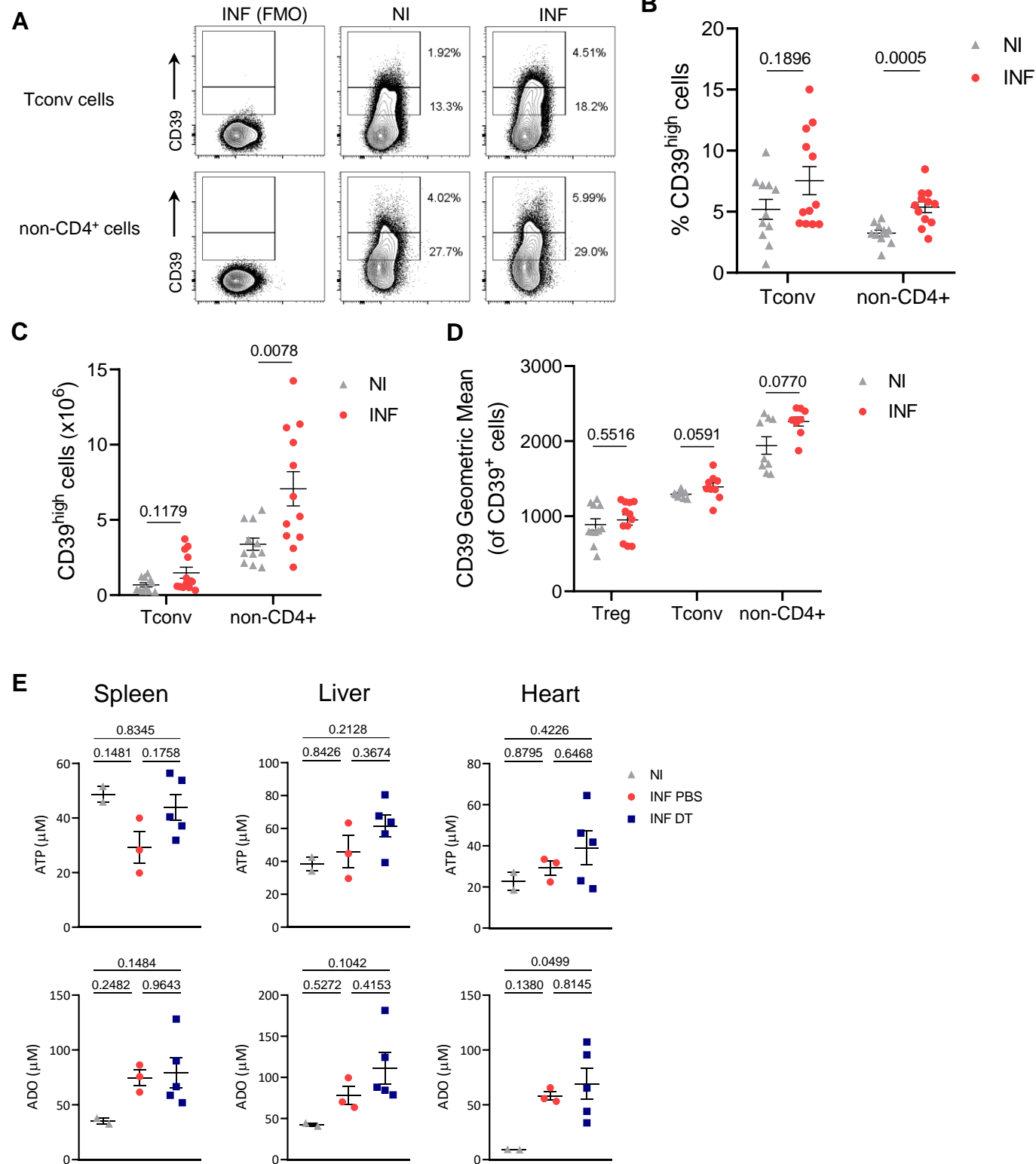

Supplement: S8 Fig — A-C) Representative flow cytometry plots (A), frequency (B) and absolute numbers (C) of CD39high Tconv and non-CD4+ cells in the spleen of DEREG mice at day 7 pi (INF) and non-infected controls (NI). D) Geometric mean of CD39 fluorescence intensity in CD39+ cells within Treg, Tconv and non-CD4+ T cells in the spleen of NI and INF (7 dpi) DEREG mice. In A-D data were pooled from 3 independent experiments and statistical significance was determined by Unpaired t test or Mann Whitney test, according to data distribution. E) Concentration of ATP and Adenosine (ADO) quantified in the supernatants of the spleen, liver and heart of NI, PBS or DT-treated INF (21 dpi) mice. Data in (E) were collected from 1 experiment. Statistical significance was determined by one-way ANOVA followed by Tukey’s multiple comparison test. All data are presented as mean ± SEM. Each symbol represents one individual mouse. P values for pairwise comparisons are indicated in the graphs. (PDF) [file ppat.1012191.s008.pdf]
