# Supplementary material for: CD39 expression by regulatory T cells participates in CD8+ T cell suppression during experimental Trypanosoma cruzi infection
Source: PLoS Pathog. 2024 Apr 29;20(4):e1012191. doi: 10.1371/journal.ppat.1012191 (PMC11081507; doi:10.1371/journal.ppat.1012191)
Supplement: S1 Table — (DOCX) [file ppat.1012191.s009.docx]

| **Supplementary Table 1. List of antibodies used for flow cytometry.** | | | |
| --- | --- | --- | --- |
| **Target** | **Clone** | **Conjugate** | **Brand** |
| CD107a | 1D4B | PE | Biolegend |
| CD11b | M1/70 | Super Bright 645 | eBioscience |
| CD11c | N418 | PE-Cyanine7 | eBioscience |
| CD127 | eBioSB/199 | PerCP-eFluor 710 | eBioscience |
| CD127 | A7R34 | PE | eBioscience |
| CD19 | eBio1D3 | APC-eFluor 780, PE-Cyanine5 | eBioscience |
| CD24 | M1/69 | PE-eFluor 610 | eBioscience |
| CD25 | PC61.5 | PE-Cyanine7, PE-eFluor 610 | eBioscience |
| CD38 | 90/CD38 | Alexa Fluor 647 | BD Pharmingen |
| CD39 | 24DMS1 | Alexa Fluor 700, PE-Cyanine7, PerCP-eFluor 710, eFluor 660 | eBioscience |
| CD39 | Duha59 | PE-Dazzle 594 | Biolegend |
| CD3e | 145-2C11 | PE | eBioscience |
| CD4 | GK1.5 | PE, APC-eFluor 780, PerCP-eFluor 710, Super Bright 645, PE-Cyanine7 | eBioscience |
| CD4 | GK1.5 | Alexa Fluor 700, APC | Biolegend |
| CD40 | 1C10 | PE-Cyanine5 | eBioscience |
| CD44 | IM7 | APC-eFluor 780, PE-Cyanine5 | eBioscience |
| CD44 | IM7 | PerCP-Cyanine5.5 | Biolegend |
| CD45 | 30-F11 | APC-Cyanine7 | BD Pharmingen |
| CD45 | 30-F11 | Alexa Fluor 700, PE-Cyanine7 | eBioscience |
| CD62L | MEL-14 | PerCP-Cyanine5.5, Super Bright 600 | eBioscience |
| CD69 | H1-2F3 | PE | BD Biosciences |
| CD69 | H1.2F3 | APC-Cyanine7, PE-Cyanine7 | Biolegend |
| CD80 | 16-10A1 | PerCP-eFluor 710 | eBioscience |
| CD86 | GL-1 | Brilliant Violet 785 | Biolegend |
| CD8a | 53-6.7 | PE, PerCP-Cyanine5.5, PE-Cyanine7, Alexa Fluor 700, PE-Cyanine5.5 | eBioscience |
| CTLA-4 | UC10-4B9 | Brillant Violet 605 | Biolegend |
| CTLA-4 | UC10-4B9 | APC | eBioscience |
| F4/80 | BM8 | APC-Cyanine7 | Biolegend |
| Foxp3 | FJK-16s | PerCP-Cyanine5.5, FITC | eBioscience |
| GITR | DTA-1 | Super Bright 600 | eBioscience |
| Granzyme A | GzA-3G8.5 | PerCP-eFluor 710 | eBioscience |
| Granzyme B | QA16A02 | APC/Fire 750 | Biolegend |
| IFN-γ | XMG1.2 | Brilliant Violet 711 | Biolegend |
| Ki-67 | SolA15 | eFluor 660 | eBioscience |
| KLRG-1 | 2F1 | PE | Biolegend |
| KLRG-1 | 2F1 | PE-eFluor 610 | eBioscience |
| LAG-3 | C9B7W | PerCP-Cyanine5.5 | Biolegend |
| LAG-3 | C9B7W | Brilliant Violet 421 | BD Biosciences |
| Ly-6C | HK1.4 | APC | eBioscience |
| Ly-6G/Ly-6C | RB6-8C5 | Super Bright 702 | eBioscience |
| MHC Class I (H-2kb) | AF6-88.5.5.3 | Super Bright 436 | eBioscience |
| MHC Class II (I-A/I-E) | M5/114.15.2 | Super Bright 600 | eBioscience |
| PD-1 | J43 | PE-Cyanine7 | eBioscience |
| PD-1 | 29F.1A12 | Brilliant Violet 421 | Biolegend |
| TIM3 | RMT3-23 | PE | eBioscience |
| TNF | MP6-XT22 | PerCP-Cyanine5.5 | Biolegend |
| TNF | MP6-XT22 | PE | eBioscience |
