# Supplementary material for: CD39 expression by regulatory T cells participates in CD8+ T cell suppression during experimental Trypanosoma cruzi infection
Source: PLoS Pathog. 2024 Apr 29;20(4):e1012191. doi: 10.1371/journal.ppat.1012191 (PMC11081507; doi:10.1371/journal.ppat.1012191)
Supplement: Attachment — Submitted filename: Araujo et al Point-by-point answer to reviewers.pdf [file ppat.1012191.s012.pdf]

PPATHOGENS-D-23-01622R1 "CD39 expression by regulatory T cells participates in CD8+ T cell suppression during experimental Trypanosoma cruzi infection"

Point-by-point response to reviewer

This manuscript presents some interesting data and the reviewers feel that the study has been well designed and the work carried out to a high standard. However, there are some concerns raised by the reviewers which require further responses before this manuscript can be considered for publication. In particular reviewer 2 has raised points about over interpretation of the data and whether the diphtheria toxin has any direct effect on T. cruzi independently of its use to regulate Treg levels. Please provide responses to the reviewers concerns (in particular those major concerns raised by reviewer 2) and it would be of advantage to directly test the effect of DT on parasites in vitro to exclude such effects from the interpretation of the results.

We sincerely appreciate the thoughtful evaluation of our paper. In response to all reviewers' comments, particularly those raised by Reviewer 2, we have carefully incorporated additional data into the revised version of the manuscript. Additionally, we made necessary revisions to moderate the conclusions, ensuring they more accurately reflect our results. We are confident that the revised manuscript is significantly improved thanks to the invaluable feedback from the reviewers. We hope it will be considered for publication in its current form.
Please, find below a point-by-point answer to all the reviewers´comments.

Part I - Summary

Please use this section to discuss strengths/weaknesses of study, novelty/significance, general execution and scholarship.

Reviewer #1: This is a very well presented and conducted study that provides mechanistic evidence on the role of Tregs on parasite control and CD8 T cell responses during acute T. cruzi infection. I do have a few comments that may improve data presentation, which I detail below (Part III -minor Issues).

We thank reviewer 1 for his/her positive comments and the feedback to improve data presentation. Please, find our response to his/her minor issues in section III.

Reviewer #2: This ms presents some interesting findings relating to a potential role for Tregs in the immune response to acute T. cruzi infection in mice. The topic is of interest and importance because there is very little understanding of why host immunity can usually control T. cruzi infection very well, but does not achieve sterile self-cure. This results in the vast majority of human infections being chronic. It is a reasonable hypothesis, given data from other systems, that Tregs could be constraining anti-parasitic CD8 T cells, which are considered to be the principal effectors against T. cruzi. Here the authors use the DEREG mouse system, which allows specific depletion of Tregs at defined time points using diptheria toxin administration. To my knowledge this is the first time this powerful experimental tool has been used to study T. cruzi infection and the authors also combine this model with adoptive transfer techniques to look more specifically at the role of CD39 in Tregs. Thus, the study could be considered fairly cutting edge for the field. The data and text are presented very clearly and for the most part, the experiments appear to have been well designed and executed.

The main weakness of the paper, in my view, is that the importance of Tregs (and expression of CD39 by them) is over-stated given the fairly limited time-frame of most of the data (most read outs are for a single time point around day 20 post-infection) and the generally small or transient effect sizes observed, particularly with respect to parasite loads.

For example, line 25, line 39, line 109 (Treg cells play a critical role), line 163 (Treg cells play a prominent role), line 276 (the crucial role of Treg cells), line 292 (Treg cells play highly relevant functions). The data do show quite clearly that depletion of Tregs results in a transient expansion of one subset of parasite-specific CD8 T cells in the liver and spleen, but this does not appear to make a very strong or consistent difference to parasite loads, perhaps 2 to 3-fold reduction in the blood and heart, sometimes a reduction in the liver (Fig 2 but not Fig 6) and no difference in the spleen around day 20-22. This might be explained by a mildly inhibitory direct effect of diptheria toxin on the parasites, which needs to be tested for. Furthermore, the data show that Treg depletion had no effect if it was initiated on day 11 instead of day 5. The data are definitely a useful contribution to the field, but without knowing whether Treg depletion makes any long term difference to the course of infection other than at this snapshot in time, or on any Chagas disease relevant pathology, it seems unjustified to claim they are critical/crucial etc.

In the title they claim that “CD39 expression by regulatory T cells drives CD8+ T cell suppression”, but this conclusion hinges on the experiment in Fig6 which involved quite an artificial system of adoptively transferring large numbers of immunosuppressive Tregs and I don’t believe there is evidence that the mechanism involves CD39 directly enough to warrant using the term “drives”. Influences or modulates might be more appropriate.

Furthermore, it is not clear how CD39 expression actually translates into higher numbers of CTLs in this system and the specific ability to respond to Tc antigen, at least in vitro, in a polyfunctional (IFNg+ TNF+ CD107a+) way (Fig 3) but not in bi- or mono-functional ways (Fig S4). For example, are adenosine levels actually different in the tissues? Is the Treg adoptive transfer effect dose-dependent and how was the number of cells (1 million) to adoptively transfer decided? What happens with T. cruzi infection in the CD39 knockout mice?

We thank Reviewer 2 for emphasizing the importance of refining the wording in our manuscript to avoid overstatement of the conclusions drawn from our results. The manuscript has undergone revisions across all sections, including the title, to temper the conclusions and ensure they accurately reflect our findings.

To address the additional comments from Reviewer 2, we incorporated new results in the revised manuscript. We conducted an in vitro assay to explicitly demonstrate that DT itself has no direct effect on parasite survival (new S2C Fig). This, in conjunction with the observation that DT injection does not impact parasitemia nor CD8+ T cell responses in WT mice (non-transgenic DEREG littermates) (new S2A and S2B Fig), supports the conclusion that the phenomenon linked to improved parasite control is specifically attributed to Treg cell depletion and not any direct effects of DT. This observation aligns with the presence of a more robust CD8+ T cell immunity in the context of Treg cell depletion.

While we acknowledge the reviewer's suggestion regarding the evaluation of long-term consequences of acute Treg cell depletion, this manuscript specifically focuses on the acute phase when the antiparasite effector response is stronger and the parasite load is dominant. A follow-up manuscript is in progress to understand the changes in the transcriptional program and tissue distribution of Treg cells that occur in the transition to the chronic phase. Preliminary results suggest that Treg

cells exhibit a different program in chronicity, accumulating in tissues where they sustain tissue repair while also influencing immune effector function. This highlights the complexities of Treg cell biology in the acute vs. chronic phase of this infection, emphasizing the need for an intensive evaluation of Treg cell biology in each infection stage.
Continuing to focus on the acute phase, we have included new results regarding the progression of the infection in terms of parasitemia, parasite load, and effector immune response in CD39 KO mice. As detailed in Section II and III, these results further reinforce the notion that CD39 pathways influence T. cruzi infection progression. Within this frame, our approach comparing the effect of Treg cell depletion followed by adoptive transfer of WT vs. CD39 KO Tregs refines this knowledge, highlighting that the expression of CD39 on Treg cells plays a relevant role in the observed phenomenon.
The Treg cell dose of $1x10^6$ cells was chosen based on a theoretical calculation and a previous study from our lab (Araujo et al, Front Immunol 2018; https://doi.org/10.3389/fimmu.2018.02555), considering the number of Treg cells required in the spleen to 'normalize' the frequency of Treg cells in that organ. While we acknowledge the inherent limitations of this theoretical calculation, implementing a dose-dependent approach, as suggested by the reviewer, presents challenges, primarily due to the considerable number of mice required for such comprehensive analysis. Given ethical constraints, we opted to use a Treg cell dose that has been previously tested in our setting. To enhance the robustness of our findings, we complemented this approach with the use of CD39 KO mice, as suggested by the reviewer.
Finally, in response to the reviewer's request, we examined ADO and ATP levels in plasma and tissues to shed light on the potential mechanisms underlying the impact of CD39 expression on CTL response and parasite load control. As depicted in Figure 6H and S8E, Treg cell depletion did not result in changes in ATP or ADO levels at 21 dpi, while the complete absence of CD39 expression, as observed in CD39 KO mice, led to increased ATP levels without affecting ADO levels (Figure 6E). In the revised version of the manuscript, we analyze and discuss these findings, considering the limitations of the selected time point for ATP/ADO quantification and the presence of a high number of cells, other than Treg cells, that express CD39 in INF mice (S8A-D Figs).

For a more detailed description of all the new results and manuscript changes resulting from Reviewer 2's revision, please refer to Section II and III.

<u>Reviewer #3:</u> In this manuscript, Furlan et al investigated the CD39+ T reg cells mediated immune suppression in early stage of experimental T.cruzi infection by regulating the CD8 T cells responses in CD39 dependent manner. Authors showed that depletion of Treg cells have impacted antigen specific CD8 T cell responses by early priming and activation of conventional CD4+ T cells Overall, this is very well written manuscripts and all the results are supported by nicely executed experiments.

We thank Reviewer 3 for the positive feedback and thoughtful evaluation of our manuscript.

Part II – Major Issues: Key Experiments Required for Acceptance
Please use this section to detail the key new experiments or modifications of existing

experiments that should be absolutely required to validate study conclusions. Generally, there should be no more than 3 such required experiments or major modifications for a "Major Revision" recommendation. If more than 3 experiments are necessary to validate the study conclusions, then you are encouraged to recommend "Reject".

Reviewer #1: No further experiments seem to be required to validate study conclusions

Thank you for acknowledging that our study's conclusions are well-supported by the presented data.

Reviewer #2: They should establish at what level diptheria toxin is directly inhibitory to T. cruzi in vitro and develop a better overview of the impact of Treg depletion on parasite load kinetics e.g. a full parasitemia curve, ideally in both the DEREG system (DT vs PBS) and for infections in the CD39 knockout mice.

Following reviewer 2 requests, the revised version of the manuscript now include:

- ***a) Results from an in vitro assay demonstrating that DT presence does not affect parasite survival in concentrations from 1.3 to 20.8 nM. These concentrations span around the concentration of 5,2 nM that is the ideal reached with the DT dose used in vivo. Calculations were performed based on approx DT MW 63kDa, dose 25 ng/g, mouse weight of 20 g and blood volume of 1,5 ml. These results are incorporated in new S2C Fig. Additionally, we have incorporated a new S1A Fig that demonstrates that in vivo injection of DT in WT mice (non-transgenic DEREG littermates) does not alter parasitemia, result that is briefly described in lines 164-169.***
- ***b) Full parasitemia curve in DEREG mice in Figure 2A.***
- ***c) Evaluation of T. cruzi infection in CD39 KO mice including full parasitemia curve that show no difference between WT and CD39 KO mice but accompanied by a significant increase in the frequency of parasite-specific CD8+ T cells (Figures 6D-F).***

Reviewer #3: Following are my comments:

1. In abstract, subsequently influencing CD8+ T cells responses (add responses).
2. Introduction, Page 4; line 60:- Immunological help
3. Introduction, Page 5; line 84-85: “Our findings…..” Please rewrite this sentence as it is not very clear.
4. Result section, Page 7; line 133-134: Please re-write the sentence “Importantly, Treg cell depletion was also observed in frequency …”

Answer to point 1-4: ***We thank the reviewer 3 for his/her suggestions to improve the redaction of the manuscript as indicated, we have revised the manuscript accordingly.***

5. Authors observed reduced parasitemia in liver and heart at day 20 p.i but not in spleen. Any explanation for this? ***The reviewer's observation regarding the differential impact of Treg cell depletion on parasitemia in different organs is indeed intriguing. The Tulahuen strain of T. cruzi has historically been considered reticulotropic, showing a preference for invading phagocytic mononuclear cells in the spleen and liver over muscle cells in the heart and skeletal muscle (10.1038/srep32927). However, variations in parasite tropism have been reported, suggesting myotropic tendencies for certain strains (10.1128/iai.67.1.403-409.1999). In our laboratory, unpublished***

data indicate that the highest parasite load is found in skeletal muscle and heart, with lower levels observed in the spleen and liver (see below). Despite parasite tropism, another factor potentially influencing parasite control in tissues is the degree of organ hyperplasia. In our experiments, INF mice displayed dramatic splenomegaly (6X weight increase), whereas the increase in liver size was less significant (1.5X increase) (see below). We hypothesize that the combination of parasite tropism, organ size increase, and variations in effector immune mechanisms between secondary lymphoid organs like the spleen and other tissues like the liver and heart may contribute to the differential response to Treg cell depletion and the subsequent increase in effector CD8+ T cell responses, ultimately impacting parasite control. A systematic evaluation of tissue parasitism in other relevant organs such as skeletal muscle, visceral adipose tissue and colon would have provided further elements to address this point. We have briefly discussed this in lines 338-342.

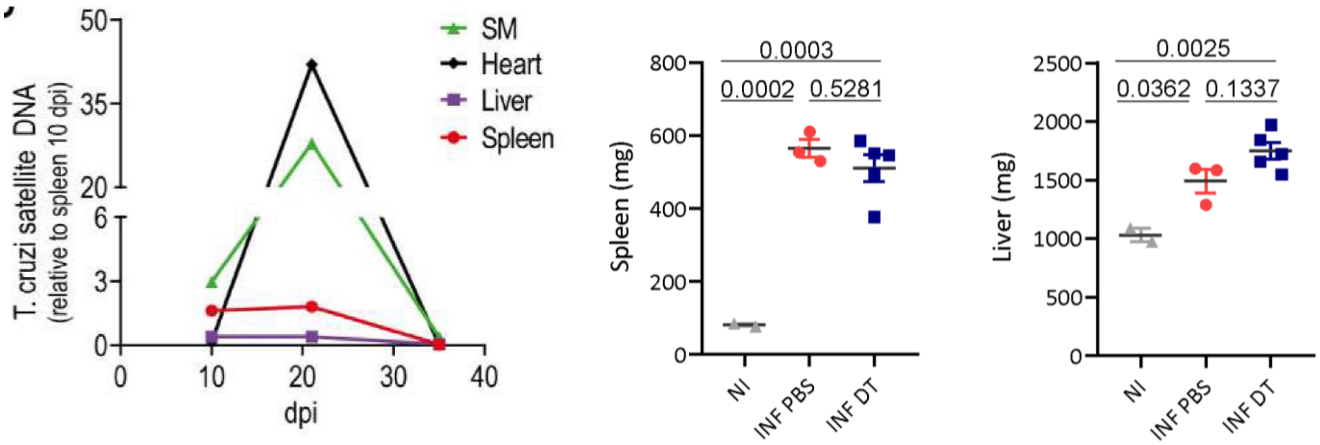

Figure for reviewer: Kinetics of parasite load determined a different days post-infeccion with T. cruzi in Foxp3-GPF mice (left panel). Weight of the spleen (middle panel) and liver (right panel) determined in non-infected (NI) and PBS- and DT-treated infected (INF, 20 dpi) DEREG mice. Statistical significance was determined by Unpaired t test or Mann Whitney test, according to data distribution.

6. Did author look at author activation markers like CD69 (early marker) and CD38 on parasite specific CD8 T cells (as shown in figure S3C and S3D Figs)?
Taking into consideration the reviewer 3 comments, we conducted a more comprehensive phenotypic characterization of total and parasite-specific T cells. This evaluation included not only other activation markers (CD69 and CD38) as suggested, but also inhibitory receptors and effector molecules. Again, no significant changes were observed in the expression of these markers when comparing total and parasite-specific CD8+ T cells from PBS-treated vs DT-treated INF mice. These results further support that Treg cell depletion primary increase the magnitude of the parasite-specific CD8+ T cell response as evidenced by the higher frequency of parasite specific cells, with a preferential increase in SLEC in certain tissues. The complete phenotypic characterization is included as new S4C figure.

7. Did author try to look the activation status of parasite specific poly-functional CD8+ T cells (that may differ compared to overall CD8+ T cells) in Treg depleted infected animals compared to control? ***Given that evaluating effector responses in vitro necessitates an activation stage with the specific parasite peptide in culture that may affect the***

phenotype of the responding cells, we did not perform additional evaluation of surface markers of activation in this setting. Instead, we conducted a comprehensive phenotypic evaluation of parasite-specific (tetramer+) CD8+ T cells as well as total CD8+ T cells in PBS-treated vs. DT-treated infected mice, as described in the previous point.

8. Page 10, line 204: Among these clusters, we observed six (?????) that displayed….. Please check and rectify the sentence. (Simply state that we observed 6 unique clusters.) ***The sentence has been rectified to improve clarity.***

9. Authors define the Treg population based on just FoxP3 expression. Did they consider using CD25 and CD127 as additional marker? ***In our previous study (https://doi.org/10.3389/fimmu.2018.02555), we extensively characterized the phenotype of Treg cells activated during T. cruzi infection. Our findings revealed that while CD25 expression is generally upregulated during infection, a significant proportion of Foxp3-GFP+ CD4+ T cells exhibit variable CD25 expression levels, ranging from high to low (refer to the extract from Supplementary Figure 4 from the published study, copied below). Additionally, our investigation showed that CD127 expression is elevated during acute T. cruzi infection, with approximately 40% of Treg cells upregulating CD127 by day 20 post-infection. Similar CD127 upregulation in activated cells has been documented in other contexts (references: 10.1002/eji.201040531, 10.1111/cei.12804). In contrast, the expression of GFP in our Foxp3 reporter mice remains consistently high and even increases during infection, enabling effective characterization of Treg cells as Foxp3-GFP+CD4+ cells.***

Supplementary figure 4

A

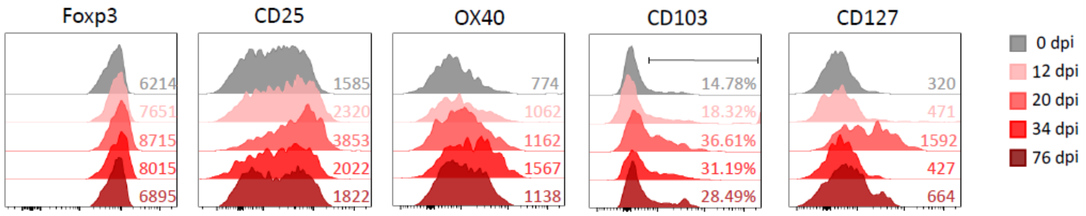

Supplementary figure 4: Expression of Treg cell markers of activation, suppressive function and Th1 specialization during *T. cruzi* infection. A) Histograms illustrating the expression of different markers of activation and suppressive function in Treg cells from the spleen of Foxp3-EGFP mice at different dpi. Numbers inside the plots indicate geometric mean of fluorescence intensity or percentage of positive cells. **B)** Dot plots showing LAP+ GARP+ double positive or IL-10 producing Treg cells

These phenotypic features of Treg cells were also observed in DEREG mice, as shown in the accompanying graphs. Therefore, we chose to identify Treg cells in our study based solely on CD4 and Fox3-GFP expression. However, we routinely include CD25 staining as a control in most of our flow cytometry experiments, with the majority of Foxp3-GFP+ T cells being CD25+, consistent with previous descriptions of this cell subset.

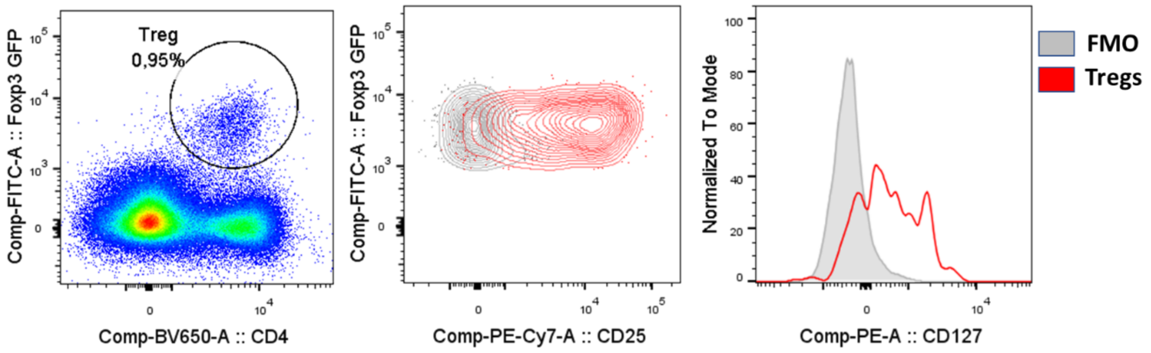

Figure for reviewer. Expression of different markers Treg cell markers determined by flow cytometry in spleen cells from *T. cruzi* infected DEREG mice at 21 days post-infection.

10. I am not much convinced with gating of CD127 and KLRG-1. Can you show FMO control (same for FoxP-3)? ***FMO control for CD127 have been added to the revised Figure 3A, and similar controls have been included in the revised Figure 6A. However, it's important to note that an FMO for Foxp3 cannot be included, as Foxp3 is not directly stained but rather identified through the surrogate presence of GFP encoded by the specific transgene in DEREG mice.***

11. Did authors also checked the CTL activity (Perforin, Granzyme etc) of CD*+ T cells in absence of Treg cells? ***Following reviewer suggestion, the expression of two molecules involved in CTL activity, such as Granzyme A and B, was assesed in total and parasite-specific CD8+ T cells from PBS- vs DT-treated INF DEREG mice and found no differences between experimental groups (see data in S4C Figure). These results go in the same direction as the absence of phenotypic differences regarding the expression of activation markers such as CD44, CD25, CD69 and CD38 and other markers analyzed. Taken together, these findings suggest that the primary effect of Treg cell depletion is on the magnitude rather than the quality of parasite-specific CD8+ T cell responses, with the exception of a slight preference towards SLEC differentiation.***

12. The quality of figure 4 (a,b & c) is not very good and convincing. Similarly Figure 6 a is not clear. ***In the revised version of the manuscript we have improved The quality of all the figures.***

13. Did authors try to block CD39 in vivo (apart from adoptive transfer experiment)? ***While we did not directly attempt to block CD39 in vivo, we addressed this aspect differently in the revised manuscript. Specifically, we investigated the progression of T. cruzi infection in CD39 KO mice, providing an indirect assessment of CD39's role. These findings are presented in Figure 6D-F.***

Part III – Minor Issues: Editorial and Data Presentation Modifications
Please use this section for editorial suggestions as well as relatively minor modifications of existing data that would enhance clarity.

Reviewer #1: 1- Presumably, authors have collected phenotypic data on Tregs from naive DEREG mice (i.e. day 0 post-infection). It would be valuable to add these data to the kinetics graphs from Fig. 1 (C and D). ***The frequency of Treg cells in NI mice treated with PBS and DT was already presented in the original Figure 1C. In response to the reviewer's suggestion, we have now included the NI experimental group in the revised Figure 1D and F.***

2- Similarly, it would be valuable to add the kinetics of blood parasitaemias for different groups (PBS and DT) to fig. 2, and not just day 20 pi as currently displayed (Fig. 2A). This would allow a visual comparison of the kinetics of blood parasitaemia vs kinetics of TSKB20+ antigen-specific CD8 T cell responses as a consequence of Treg depletion (i.e. Same time points for Fig. 2 A and D). ***The complete kinetics of blood parasitemias is now incorporated into the revised Figure 2A.***

3- Fig 3, it would be valuable to show the FMO control for the CD127 staining. ***FMO control for CD127 has been added to the revised Figure 3A.***

4- Fig 3, the impact of Treg depletion on antigen-specific CD8 T cell responses are very limited in terms of absolute counts (i.e. only counts of TSKB20+ SLECs in the liver are affected). Authors should further discuss this apparently limited impact. ***We respectfully disagree with the characterization of the impact of Treg depletion on antigen-specific CD8 T cell responses as limited. In Figure 2D-E, we observed a doubling in the frequency of specific CD8 cells at several time points in the blood, as well as at 20 dpi in the spleen and liver. This doubling results in a significant increase in the number of parasite-specific CD8 cells in both organs (Figure 2F). Considering that CD8+ T cell immunity arising during T. cruzi infection is well known to be polyfunctional and effective in controlling parasite load in different experimental models (reviewed by us in https://doi.org/10.1016/j.pt.2019.08.006), and that this phenotype is conserved upon Treg cell depletion, the observed increase in CD8+ T cells may certainly have an impact on parasite control.***
In addition to the evaluation of the magnitude of parasite-specific CD8+ T cell responses, we performed a phenotypic characterization of the parasite specific CD8+ T cells arising in the context of Treg cell depletion. Our analysis first focused on differentiation subsets defined by CD127 and KLRG1 within the CD8+ T cell pool, which revealed an enrichment in SLECs in terms of frequency in both the spleen and liver that resulted in increased SLEC numbers only in the liver, but not the spleen (Figure 3B-C). However, this result does not preclude the possibility that other subsets not included within the SLEC and MPEC classification are those enriched within the specific CD8+ T cell pool and may play a significant role. In response to the reviewer's comment, we have revised the description of the results and provided additional discussion in the revised manuscript to further clarify this point.

5- Fig 6A, contour plots with outliers instead of density plots will probably be a better representation for this data. Actually, replacing density plots with contour plots (or zebra plots) throughout the manuscript will probably improve flow data presentation. In addition, displaying FMO controls for CTLA-4, CD25 and CD39 in this figure would be of value. **In**

response to the reviewer's suggestion, we have incorporated the FMO in the revised Figure 6A. Additionally, we have replaced density plots from various figures with contour plots to enhance data presentation.

6- Fig 6B, what about absolute counts? Could authors please display absolute counts side-by-side % in Fig 6B? ***In accordance with the reviewer's recommendations, we have added absolute counts of CD25+, CTLA4high, and CD39high Treg cells as a new Figure 6C.***

7- Fig 6E, have authors checked for viability of cells before adoptive transfer? Please clarify and display data if possible. ***Transferred iTregs were typically 80% live cells (Aquadead negative) and 90% Foxp3 positive, as shown below. A brief mention of these aspects has been added to the revised Material and Methods Section in lines 564-565.***

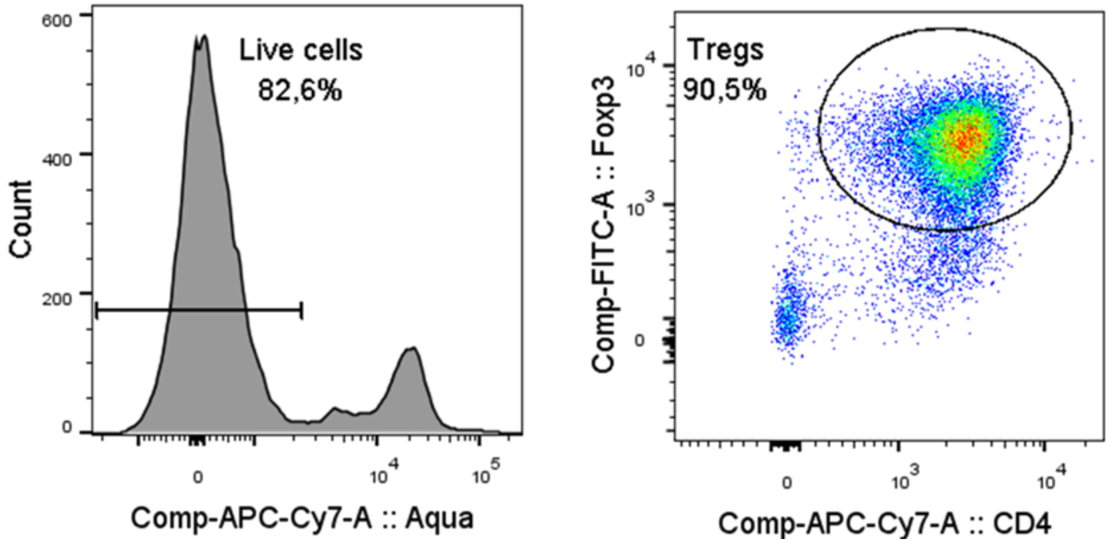

Figure for reviewer: Percentage of live cells as determined by Aquadead staining (left) and percentage of Treg cells as determined by Foxp3-GFP and CD4 expression after differentiation of in vitro Treg cells for adoptive transfer experiments from Figure 7.

8- Fig 6E, authors mention they used in vitro splenocytes from CD39 KO mice to generate KO iTregs. However, the CD39 KO mouse strain used in the study is not mentioned in M&M. Please, add this information. ***We appreciate the reviewer for bringing this point to our attention. In the revised version of the manuscript, we have addressed this omission and provided a complete reference of the CD39 KO strain used.***

9- Authors suggest CD39 as a potential target for immunomodulatory therapeutic strategies during T. cruzi infection. However, authors performed this study only during acute infection, and effects were only detected following very early interventions (i.e. 5-6 days pi) after primary infection. Strong word of caution here; it is extremely unlikely for clinicians to intervene very early after primary T. cruzi infection in humans. In any case, even if very early intervention was possible (e.g. in the case of a laboratory accident), the preferred avenue for treatment will certainly be benznidazole, for which there is strong clinical evidence of its benefits during acute infection. Therefore, I would recommend not to suggest CD39 as a potential therapeutic strategy at least until after authors are able to

collect meaningful data during chronic experimental infections. ***We appreciate the reviewer's insightful analysis and agree with the importance of considering the clinical implications of potential therapeutic strategies. It is indeed crucial to prioritize pharmacological treatment, such as benznidazole, especially during acute T. cruzi infection, for which there is strong clinical evidence of its benefits.***
We acknowledge the limitations of our study, which focused on acute infection and involved early interventions. We agree that suggesting CD39 as a potential therapeutic strategy should be approached with caution, particularly without data from the chronic stage. Although we have been able to demonstrate the role of CD39 expression on Treg cells in regulating CD8+ T cell immunity and parasite control during the acute phase, and have added new data using CD39 KO mice, further research is needed to assess the effects of CD39+ Treg cell depletion during the chronic phase of infection before considering this avenue of treatment. Accordingly, we have revised the discussion section to emphasize the need for caution and further evaluation before targeting the CD39 pathway as a possible therapeutic strategy for T. cruzi infection.

<u>Reviewer #2:</u> 1. For some data sets the legends state that data are pooled from independent experiments while for others the authors show data from one experiment and state that similar results were obtained in independent experiments. How did they decide when to pool data and when to not pool? Does “similar results” mean that the same statistically significant differences were reproduced in all cases? ***Reviewer #2 raises a valid point regarding the pooling of data from independent experiments in our manuscript. The majority of the data presented were pooled or collected from different independent experiments, encompassing infection experiments in both wild-type and knockout mice, as well as experiments with diphtheria toxin (DT) treatment. In these cases, we calculated the sample size required to achieve statistical significance and divided these numbers across 1-3 independent experiments.***
However, there were three exceptions where we presented data from representative experiments. Firstly, for the in vitro evaluation of CD8+ polyfunctionality, due to the specific features of the in vitro approach, it was necessary to show each experiment independently. Secondly, in the unsupervised analysis of APC distribution and phenotype in Treg cell-depleted vs. non-depleted INF DEREG mice, due to the extensive amount of data analyzed and the features of UMAP algorithms, it was not feasible to pool data from different experiments. Lastly, for the adoptive transfer experiments involving iTreg differentiation, cell transfer, infection, and DT treatment, we opted to manage them in two independent experiments and showed one representative.
We appreciate the reviewer's inquiry regarding the interpretation of "similar results." Indeed, "similar results" indicate that the same statistically significant differences were reproduced across independent experiments. We have further clarified this point in the Material and Methods section (Lines590-594) of the revised manuscript.

2. Lines 59-61: The three papers cited here are all on immune responses in mice and come from the same lab – have these phenomena been shown to be reproducible in human studies?
3. Line 81-88: Should clarify that the study discussed relates to mice. In general, the introduction would benefit from a stronger distinction between mouse and human data, even if it means highlighting where there are gaps regarding human infections.

Answer to comment 2 and 3. We appreciate the reviewer's feedback on the need for a clearer distinction between mouse and human data in the introduction section. We agree that emphasizing this difference is crucial for accurately contextualizing the research findings. To address this, we have made revisions to clarify that the studies discussed pertain specifically to murine models. Additionally, we have incorporated additional references from both murine and human studies to strengthen the significance and reproducibility of the phenomena described.

4. Figure 1C,D,E: It is difficult to interpret these figures because the data are presented as the frequency of Tregs within the total CD45+ population, which is going to be massively affected by increasing frequencies of other cell types e.g. CD8 T cells, as a natural consequence of the infection. It would be clearer to present the frequencies as % of total CD4+ cells rather than/as well as CD45+ cells. The authors do present total numbers of Tregs, but only for spleen and liver at one time point (day 20) and here the depletion is significant, but with quite a small effect size. ***We respectfully disagree with the reviewer's claim that Treg cell frequency within the CD4+ subset instead of the total CD45+ population is clearer. We are convinced that presenting Treg cell frequency within the total CD45+ population provides a more comprehensive view of their regulatory role, as Treg cells can modulate various immune cell populations within this subset, including B cells, CD4 cells, antigen-presenting cells, and CD8 cells. Analyzing Treg cell frequency within the complete pool of cells they may regulate seems more relevant than evaluating it solely within the CD4 pool. Even if the reduction in Treg cell frequency is influenced by an increase in other subsets, it still indicates that the regulatory capacity of Treg cells is overwhelmed by an increased presence of cells that they are supposed to regulate. However, to address the reviewer's concern, we have included the Treg cell frequency within the CD4+ subset in the revised version of our manuscript as supplementary data (S1A and S1B Figure).***
Regarding the presentation of total Treg cell numbers, we agree with the reviewer that showing the numbers only at 20 dpi exhibits a small effect size. Therefore, we have included a kinetics analysis of these values in the revised manuscript, demonstrating a clear reduction in Treg cell numbers from 7-8 dpi onwards, which remains significant even at 20 dpi when natural Treg cell contraction is maximal (see new Figure 1F).

5. Related to this, was there a difference in spleen and liver weight between the PBS and DT-treated infected groups? Was this taken into account?
We appreciate the reviewer's inquiry regarding potential differences in spleen and liver weight between the PBS and DT-treated infected groups. While we did weigh the organs in a few experiments and observed no significant differences (see below), we acknowledge that this assessment was not conducted systematically across all experiments. However, it is important to note that we systematically count total leukocyte numbers in all experiments, which are then used to calculate the number of specific subsets as depicted in several figures. Therefore, we have a quantitative method in place to account for any change in the overall leukocyte presence in the spleen and liver between the PBS- and DT-treated infected groups. This methodological approach has been further clarified in the Material and Methods section, specifically in the Cell Preparation subsection.

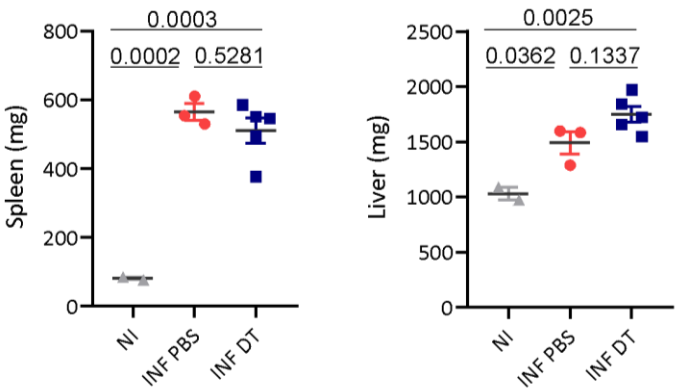

Figure for reviewer. Spleen and liver weight of non-infected (NI) and PBS- and DT-treated infected (INF, 20 dpi) DEREG mice. Each symbol represents one mouse. Statistical significance was determined by one-way ANOVA followed by Tukey's multiple comparisons test .

6. Figure 2B and 6G: y axis labels should be relative to GAPDH not PBS? Also, why not show all the data points as in most other figures? ***The y-axis labels indicate relative fold change of gene expression compared to PBS-treated mice, as calculated using the ΔΔCT algorithm. This method includes GAPDH as a housekeeping control and uses the value of T. cruzi DNA in PBS-treated mice as a reference sample. As established in our laboratory (10.3389/fimmu.2017.01548; 10.3389/fimmu.2018.02555; doi/10.1128/mbio.00447-20) and described in the Material and Methods section, tissue parasitism is evaluated in pooled tissues from the mice of each group in each independent experiment. Therefore, we consider it more appropriate to present the data using bar graphs with error bars.***

7. Figure 2C: needs a label (or edited legend) to indicate what are these cells gated on. ***The legend "CD45 gated" has been included in the revised Figure 2C for each of the tissues evaluated***.

8. Figure 2D,E: it would be useful to see the same quantification data for tetramer-negative CD8 T cells, many of which will still be specific for other parasite antigens. ***Following the reviewer's suggestions, we have incorporated new figures S1C, S1D, and S1E, illustrating the frequency and absolute number of total CD8+ T cells following DT injection. These findings demonstrate that the frequency of the total CD8+ T cell population increased in blood and liver but not in the spleen upon Treg depletion. Additionally, total CD8+ T cell counts remained overall unaffected by Treg cell depletion. These results are described in lines 159-163.***

9. Line 149: “the observed effects on the T. cruzi-specific CD8+ T cell response and parasite levels were not attributable to potential toxic effects of DT itself but a consequence of Treg depletion, as they were not detected in DT-treated WT littermates (S1A Fig).” The inclusion of “parasite levels” here is not supported by the data, only T cell frequencies were measured. The authors should have tested the direct effect of DT on T. cruzi e.g. using an in vitro assay. ***To address the concern raised by reviewer 2, we conducted additional experiments and included two new pieces of data in the revised S2 Figure. Firstly, we added the missing data on blood parasitemia in PBS and DT-treated WT littermates, demonstrating no effect on parasite levels at day 20 pi (S2B Figure). Additionally, we performed an in vitro experiment to evaluate the direct effect of DT on T. cruzi viability. This experiment assessed a wide range of doses, including those used in vivo, and revealed no direct effect of DT on parasite***

viability (S2C Figure). These new findings support our conclusion that the observed effects on parasite load are not a consequence of direct toxic effects of DT on the parasite, but rather due to Treg cell depletion, resulting in an enhanced T. cruzi-specific CD8+ T cell response. A brief reference to these results has been included in the results section, lines 164-169.

10. Line 157: “the time point [day 11+12] when the natural contraction of the Treg cell response begins”. Data are not shown to demonstrate the kinetics of this “natural contraction” – as noted above, the change in frequency is mainly a result of increasing numbers of other cell types, such as CD8 T cells. Reference 13 should probably therefore be cited here because it did show some changes in total Treg numbers over time, but it is not clear from the description of the statistical analysis of those data (Figure 1 in that paper) it what sense the contraction was significant. ***As mentioned in our response to reviewer 2 comment 4, we maintain our stance that the significant reduction in the frequency of Treg cells within the CD45+ population is of physiological significance. This reduction implies that the regulatory response may be overshadowed by the expansion of various subsets of effector immune cells. However, we acknowledge the reviewer's concern regarding the terminology used and the need for referencing our previous work to support our statement. We have made the necessary revisions to clarify the terminology and have added a reference to our previous work on Treg cell responses and effector CD8+ T cell responses for additional context (lines 176-178).***

11. Line 179: “the increase in the SLEC subset was the primary consequence of Treg depletion on CD8+ T cell responses at day 20 pi” the sentence should be qualified that the finding only applies to the liver and not the spleen. ***We appreciate the reviewer's comment pointing out that our description was not accurate, which is somewhat linked to comment 4 from reviewer 1. As discussed previously, the most significant effect of Treg cell depletion is an increase in the magnitude of CD8+ T cell responses, characterized by an increase in the frequency and numbers of total and parasite-specific CD8+ T cells in the spleen and liver. Within the increased total and parasite-specific CD8+ T cells, we observed an enrichment of SLEC cells in terms of frequency in the spleen and liver, which correlated with increased numbers only in the liver. We have introduced text edits in several parts of the results section (see lines 197-204 and 213-251) to describe the data more accurately and clarify the conclusions drawn from them.***

12. Line 199-201 / Figure 4: Were the non-infected mice given PBS or DT or nothing? Why wasn’t the effect of DT vs PBS tested in these control mice? Text and figure legend needs to be edited to make it clearer what the non-infected controls were given. ***In Figure 4, non-infected (NI) mice were given PBS. We did not test PBS vs DT in non-infected mice to limit the use of experimental mice, as the main aim of the NI group in this experiment was to serve as a control to define antigen-presenting cell (APC) populations rather than to test a possible effect of short term Treg cell depletion. DEREG mice are bred in a heterozygous scheme, so obtaining enough mice for the different groups requires a larger number of mice, resulting in many WT littermates being generated and not necessarily used. In this context, we concluded that it was not necessary for our scientific objective to include a group of DT-treated NI mice to evaluate the early impact of Treg cell depletion on APC biology under homeostatic conditions. Instead, we focused on understanding this early effect in the context of***

an increasing inflammatory response during early infection. We clarified the PBS treatment in NI controls as suggested by the reviewer.

13. Line 206/Figure 4: It would be easier if the cell type names were used in the figures instead of the cluster number codes. ***Following the reviewer's suggestion, in the revised version of Figure 4C and 4D, we have replaced the cluster numbers with the corresponding cell type names.***

14. Line 224: “At day 20 pi, animals that received PBS or DT on days 5 and 6 pi showed similar frequencies of Tconv cells in blood, spleen, and liver (S6A Fig).” The data in S6B are not mentioned even though they show the total numbers in the liver are significantly decreased at day 20. ***We appreciate the reviewer's attention to the lack of description of the data presented in S6B Fig, which now forms part of the new S7 Fig. This data, illustrating significant decreases in total numbers in the liver at day 20 pi, has been incorporated into the Results section accordingly.***

15. Figure 6A,B and text: how was the gating of CD39 int and high expression set? It would be useful to show an FMO control for this antibody and to show the statistical comparison of mean fluorescence intensity. ***The stratification of Treg cells based on CD39 expression into CD39hi and CD39int populations was guided by our previous research, where we demonstrated gradual upregulation of CD39 in CD8+ T cells infiltrating tumors. CD39hi and CD39int CD8+ T cells exhibit distinct phenotypic and functional characteristics, with the former displaying higher activation, increased expression of inhibitory receptors, and enhanced ATP hydrolysis capacity (DOI: https://doi.org/10.1158/0008-5472.CAN-16-2684). Similar distinctions in CD39 expression within CD8+ T cell subsets have also been observed during viral infections (DOI: 10.1371/journal.ppat.1005177). Additionally, variations in CD39 expression within Treg cell subsets, particularly in human samples, have been documented (DOI: 10.1038/cmi.2016.30, 10.3390/ijms241914442).***

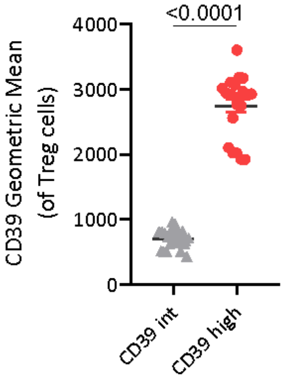

In our flow cytometry analysis, we identified CD39hi and CD39int Treg cell populations using a gating strategy that relied on a fluorescence minus one (FMO) control to determine background staining levels. We then established gates based on the observed staining shape and distribution in experimental samples compared to the FMO control. Additionally, we validated our gating approach by assessing the statistical significance of the differential geometric mean of expression between the defined populations (see graph).
In response to the reviewer's suggestion, we have integrated FMO staining for CD39 and all markers from Figure 6. While we maintain CD39high Treg cells as the focus in the main figures (Fig 6B, C), we have included data on MFI of CD39 staining in Treg cells and other cell subsets in the new supplementary figure S8C.

16. Figure 6E,F: In some cases there appear to be fewer data points for the absolute counts compared to the frequencies e.g. PBS group spleen and DT+KO Treg group for spleen and liver. ***We appreciate the reviewer's attention to the discrepancy in the number of data points between frequency and absolute numbers in Figure 6E and F. This disparity arose due to the removal of outliers in TSKB20+ CD8+ T cell counts in***

the spleen and liver of mice from two experimental groups. It's important to note that these experiments already have a limited sample size due to the nature of the study. For this experiment, each data point demands a significant number of animals including those required to obtain WT and KO iTregs that are adoptively transferred, and the controls.
Given ethical considerations, sample size was kept to the minimum possible. As a result, the removal of outliers inadvertently reduced the sample size even further. Considering this, in the original version of the manuscript, we removed the outlier only in the graph of cell counts where the ROUT method detected the outliers and not in the frequency graph where the distribution was adequate. Given the reviewer's comment, we decided to remove all the data from those mice. Despite the reduction in the number of experimental points, it's crucial to emphasize that observed differences in the frequency graphs remained significant. Importantly, this change allows us to ensure consistency across experimental groups and maintain the integrity of the data.

17. There looks to be a much larger effect of DT-mediated Treg depletion on the CD8 T cell compartments vs PBS control (the groups without adoptive Treg transfer) in this experiment compared with Figure 2F and 3B. In figure 6, the splenic parasite-specific T cells (total and SLECs) are increased more than 10-fold compared to around 2-fold in the earlier data. The SLEC numbers go from being not-significantly different to highly significantly different. Maybe this just reflects normal inter-experiment variability, or perhaps there was something different in the methodology to explain this? It deserves to be commented on anyway. ***We acknowledge the reviewer's observation regarding the increased effect, particularly in SLEC numbers, when comparing the results depicted in Figure 7 to those in Figures 2 and 3. The only methodological difference between the experimental data presented in Figure 2F, 3B, and the previously Figure 6E and 6F (now 7) is that the endpoint was 22 dpi instead of 20 dpi. While this 2-day difference in infection progression might contribute to a stronger effect after Treg depletion, it is also possible that the observed difference is due to inter-experiment variability. However, considering that the direction of the results remains consistent and the data in Figure 7 serve as controls rather than the main focus, we believe it is unnecessary to comment on this discrepancy in the manuscript.***

18. Figure 6G: Legend does not state sample sizes for this experiment or if the results were reproducible in independent experiments. Also, it would have been useful to see the effect on blood parasitaemia (definitively live parasites) in this experiment as in Fig 2A. ***We appreciate the reviewer's attention to this omission. Similar to Figures 6E and 6F, the results presented in Figure 6G from the original manuscript were also representative of 2 independent experiments. In the revised manuscript, results from Figure 6C-G are included in the new Figure 7, and information about the number of independent experiments has been added to the corresponding legend: "Results similar to (C-F) were obtained in 2 independent experiments" (Line 1058). Regarding the suggestion to incorporate blood parasite quantification in the adoptive transfer experiment, we have included this data in new Figure 7E. We observed no difference in blood parasitemia upon depletion followed by the adoptive transfer of WT vs. CD39 KO Tregs, indicating that the effect was restricted to parasite load in tissues. We have also briefly discussed these results in the revised manuscript, lines 311-314.***

19. Line 262: “Importantly, T. cruzi load in spleen and liver was significantly reduced when

Treg-depleted mice were transferred with CD39 KO iTreg cells compared to mice that received WT iTreg cells” I disagree with this interpretation, the parasite load was not “reduced” in those mice by the treatment (there was no significant difference vs the PBS or DT-only control), they were protected from the parasite-load boosting effect that comes from adoptively transferring large numbers of immunosuppressive cells. It is still a convincing result in terms of the immunosuppressive phenotype of the Tregs being CD39-dependent, at least in that particular experimental setting. The same point applies to the statement in the abstract (line 30) that “the absence of CD39 expression on Treg cells … [led to] to improved parasite control during T. cruzi infection” – just saying control improved during T. cruzi infection does not capture the full setting, which was T. cruzi infection plus adoptive transfer of Tregs. ***Thank you for your valuable insight. In response to your feedback, we have revised the sentence "Importantly, T. cruzi load in spleen and liver was significantly reduced when Treg-depleted mice were transferred with CD39 KO iTreg cells compared to mice that received WT iTreg cells" to read "Importantly, we observed a significant increase in T. cruzi load in spleen and liver when Treg-depleted mice were transferred with WT iTreg cells, compared to those receiving CD39 KO iTreg cells." Additionally, we have updated the Abstract with the revised version reading: "Crucially, the absence of CD39 expression on Treg cells significantly bolstered effector parasite-specific CD8+ T cell responses, preventing increased parasite replication in T. cruzi infected mice adoptively transferred with Treg cells."***

20. Line 286: “we found that removal of endogenous Treg cells shortly after infection, but not at later stages, improved parasite control” It should be acknowledged that this was not always the case e.g. Fig 2B spleen and Fig 6G spleen and liver. ***We appreciate the reviewer's attention to the nuanced findings regarding early Treg cell depletion and parasite control. While we agree that this effect was not consistently observed across all tissues, our data clearly demonstrate improved parasite control systematically, as underlined by reduced parasitemia, as well as diminished parasite load in the liver and heart following early Treg cell depletion, as shown in Fig 2A and 2B. However, we acknowledge that this effect was not replicated in the spleen, as indicated in the same figure. Additionally, in Fig 6G, although the PBS and DT groups had a reduced number of replicates, the trend of the results remained consistent, with similar parasite loads in the spleen of both groups and a reduced load in the liver of the DT group compared to the PBS group. Therefore, we maintain that early Treg cell depletion resulted in improved parasite control in certain tissues, including the liver and heart, while acknowledging the lack of consistency in the spleen. Accordingly, we have amended the text to reflect these findings: 'we found that removal of endogenous Treg cells shortly after infection, but not at later stages, improved parasite control in blood as well as in certain tissues”, lines 337-338. In addition, we have included a brief discussion on these findings (lines 338-341).***

21. Line 389: Information is missing on nature of CD39 KO mice. ***The description of the CD39 KO strain was included in the revised manuscript. Line 462.***

22. Line 395: How were trypomastigotes purified from blood to prepare the inocula in PBS? ***The infection protocol in our laboratory does not involve purifying parasites from blood to prepare the inocula in PBS. Instead, the infective dose is obtained by diluting the blood obtained after parasite passages. Typically, the required dilution***

is around 400X. This has been further clarified in the Material and methods section. Lines 471-472.

23. Line 436: BV412, should be 421? ***Effectively, the fluorochrome was BV421 and the mistake has been corrected in the revised manuscript.***

24. Line 432: Flow cytometry methods reporting does not include details of how gating co-ordinates were decided e.g. isotype or FMO controls. ***We appreciate the reviewer's attention to detail regarding the flow cytometry methods. Gating coordinates were determined using negative controls for population markers, where staining was carefully titrated during antibody mix setup, and fluorescence minus one (FMO) controls for activation markers, regulatory molecules, or continuously expressed molecules. We have now included this information in the Material and Methods section, under the flow cytometry subsection. Additionally, FMO controls for certain staining, including CD127, CD25, CTLA4, and CD39, have been included in the corresponding figures.***

25. Line 487: "Outliers were identified using the ROUT method" – in which analyses where outliers actually removed and which statistically significant differences were contingent on removal of outliers? ***Outliers were routinely identified using the ROUT method in all datasets before conducting statistical analyses of differences. This procedure has been outlined in the Material and Methods section. While this approach may occasionally lead to achieving statistical significance, especially in experiments with small sample sizes, it's important to note that the potential impact of outlier removal on statistical significance was not systematically assessed in each dataset.***

26. Line 819: "A total of 2-18 mice per group were included (minimum 2 mice/group/experiment)" This is a very large variation in sample size and it would be better to give more details about which data points are supported by many e.g. 18 vs few e.g. 2 mice. ***In response to the reviewer's suggestion, we have provided additional details regarding the number of replicates for each piece of data.***

Reviewer #3: N/A
